# A Lost Art

**Published:** 1875-08

**Authors:** 


Efficiency of Mineral Water in Reducing and Adding Fat.—Perlet, the well-known actor, whese leanness is described as "something phenomenal," naturally desiring to get "some flesh on his bones," a well-known physician of Paris advised him to go to one of the bathing places in the Pyrenees. Perlet accordingly went off to the prescribed locality, where he drank and bathed with the utmost zeal and perseverance. But neither drinking nor bathing seemed to have any effect upon him, and he remained just as much a skeleton as before. "Patience!" urged the local doctor, in reply to his expressions of disappointment; "there is nothing like the water of our springs for making people fat!" Soon after Perlet was patiently soaking himself in a bath, and heard a colloquy in the bathing cabinet next his own between the local Æsculapius and a lady of enormous obesity. "Doctor," remarked the lady, "I am really losing heart and patience." "Why so?" inquired the doctor. "Because, though I have been taking these waters regularly for two months, I am not one ounce lighter!" "Patience, madam!" said the doctor, in his most persuasive tones, "*there is nothing like the water of our springs for making people thin!*"—*The Doctor.*

